# Faster, more accurate, more confident? An exploratory experiment on soccer referees’ yellow card decision-making

**DOI:** 10.3389/fpsyg.2024.1415170

**Published:** 2024-07-31

**Authors:** Hongbiao Wang, Chenping Zhang, Zhiguang Ji, Xiawen Li, Liyan Wang

**Affiliations:** ^1^Department of Physical Education, Shanghai University of Medicine and Health Sciences, Shanghai, China; ^2^College of Rehabilitation Sciences, Shanghai University of Medicine and Health Sciences, Shanghai, China

**Keywords:** decision-making, soccer, referee, yellow card, overconfidence, analytical decision-making, intuitive decision-making

## Abstract

This study aimed to examine how soccer referees make decisions about issuing yellow cards for fouls. The research involved 60 male participants, divided into expert (*n*=30) and novice (*n*=30) groups based on their experience and qualifications as referees. They took part in a 2×2×2 mixed-design experiment. The study looked at Decision-Making Style (DMS: Analytical Decision-Making [ADM] vs. Intuitive Decision-Making [IDM]), Video Type (yellow card foul vs. non-yellow card foul), and Referee Level (expert vs. novice) as independent variables. The dependent variables were accuracy rate (ACC), discrimination index (D), self-confidence index (C), and overconfidence index (OC). The findings showed that Analytical Decision-Making (ADM) led to higher accuracy compared to Intuitive Decision-Making (IDM). Expert referees demonstrated better accuracy than novice referees. There was also an interaction between Decision-Making Style and Referee Level, showing differences in the effectiveness of ADM and IDM between expert and novice referees. Additionally, the study revealed that both expert and novice referees showed overconfidence, with experts demonstrating significantly higher overconfidence, particularly during IDM. In conclusion, the research highlighted the complexity of referees’ decision-making in high-pressure situations and emphasized the potential benefits of employing Analytical Decision-Making strategies. The study contributed to understanding cognitive biases in sports officiating and suggested the need for targeted training programs to help referees improve their performance and reduce overconfidence in challenging situations.

## Introduction

Decision-making (DM) is critical to a football referee’s responsibilities. On the field, when faced with complex foul situations, referees must make swift and accurate decisions. The swifter and more precise the DM, the smoother the flow of the game. Given the complexity of making good and quick DM, it comes as no surprise that referees demonstrate a relatively high rate of DM errors (Catteeuw et al., 2010). Numerous studies have highlighted the accuracy rate (ACC) of referee foul decisions, spanning from 50 to 93.1% (MacMahon et al., 2007; Catteeuw et al., 2009; Mascarenhas et al., 2009; Schweizer et al., 2011; Mallo et al., 2012; Spitz et al., 2016, 2018; Jochim et al., 2018; Hossner et al., 2019). In football DM, the Union of European Football Associations (UEFA) Refereeing expert panel’s ACC in actual matches is 70% (Fuller et al., 2004). Additionally, Gilis et al. (2007) conducted a retrospective video analysis of referee performance during the 2002 FIFA World Cup in Korea/Japan, revealing that referees made correct decisions in 60% of player-to-player contact fouls. This implies that in some studies, the ACC of football foul decisions is almost equivalent to flipping a coin (Samuel et al., 2021).

Given the considerable rate of erroneous DM behavior exhibited by referees, numerous studies have delved into the mechanisms and influencing factors underlying referees’ DM. In terms of DM mechanisms, these include sequential effects (Plessner and Betsch, 2001), heuristic DM (Hepler and Feltz, 2012; Raab, 2012; Ramanayaka et al., 2023), stereotypes (Jones et al., 2002; Van Quaquebeke and Giessner, 2010), unwritten rules (Plessner and Raab, 1999; Raab et al., 2019a,b), and priming effects (Ste-Marie, 2003). As for the influencing factors of referees’ DM, they primarily involve individual experience factors and match environment factors (Lane et al., 2006). Individual experience factors mainly include referees’ physical fitness (Castagna et al., 2007; Castillo et al., 2019; Bouzas-Rico et al., 2022; Castillo-Rodríguez et al., 2023), visual skills (Pizzera and Raab, 2012), attention (Pietraszewski et al., 2014), stress coping (Wolfson and Neave, 2007), self-confidence (Çar et al., 2022), self-control (Samuel et al., 2018), expertise experience (MacMahon et al., 2007; Gilis et al., 2008; Catteeuw et al., 2009; Dawson, 2012), referee height (McCarrick et al., 2020), and self-efficacy (Guillén and Feltz, 2011). Regarding match environment factors, these include home advantage (Goumas, 2014; Lovell et al., 2014; Nevill et al., 2017; Picazo-Tadeo et al., 2017), the referee’s position on the field (Mallo et al., 2012), player complaints after fouls (Lex et al., 2015), team uniform color (Krenn, 2014; Picazo-Tadeo et al., 2017), weather (Gaoua et al., 2017), height of the fouler (Van Quaquebeke and Giessner, 2010), team reputation (Jones et al., 2002), team ranking (Castillo et al., 2018), match time (Lago-Peñas and Gómez-López, 2016), and the distance of the audience from the pitch (Dohmen, 2008).

Despite the extensive research on the DM mechanisms and influencing factors of referees, which has generated significant findings (Bar-Eli et al., 2011) and enhanced our understanding of referees’ DM (Raab et al., 2019a,b), there is a noticeable absence of studies on referees’ decision-making style (DMS) within the context of DM mechanisms. Similarly, in the domain of personal influencing factors, there have been no reports on the issue of overconfidence in referees’ DM.

Existing research has modeled the information processing of football referees’ DM behavior (Plessner and Haar, 2006), suggesting that DM actions follow the cognitive process of stimulus-perception-categorization-memory-integration-behavioral response. Consequently, errors in referees’ DM may stem from minor inaccuracies at different steps within the information processing sequence, and the probabilistic nature of Intuitive Decision Making (IDM) may serve as a significant source of error in penalty DM. The perspective on DMS posits that human judgment and DM are the result of the interaction between two distinct cognitive systems (System 1 and System 2) (Kahneman and Frederick, 2002). System 1 engages in intuitive, heuristic, automatic information processing, while System 2 engages in analytical, deliberate, and controlled information processing (Meyer and Frederick, 2023). For referees, the rapidity of DM may rely more on System 1’s IDM, whereas the accuracy of DM may depend on the deliberate Analytical Decision Making (ADM) of System 2. However, it remains unclear how the two systems switch and operate in parallel. Some studies have indicated that when contextual cues (such as previous penalty DM) cast doubt on the initially triggered DM, they prompt deliberate and slower System 2 DM (Helsen et al., 2019). Therefore, given the characteristics of referee situation problem-solving, DM in refereeing sports contexts tends to be dominated by System 1 processing patterns, supplemented by System 2 processing patterns, adhering to a dual-system processing paradigm. Consequently, IDM becomes the primary form of DM in refereeing sports contexts, with rapidity, probability, and directness becoming the fundamental characteristics of referees’ sports IDM (Araújo et al., 2019).

Overconfidence is when an individual is overly optimistic about their knowledge, abilities, or judgments (Alpert and Raiffa, 1982). Self-aggrandizing individuals tend to overestimate their accuracy and control, underestimate risk and uncertainty, and ignore or insufficiently consider information that contradicts their views (Hoffrage, 2022). While overconfidence can foster ambition, determination, perseverance, morale, and the credibility of bluffs, it can also lead to flawed assessments, unrealistic expectations, and risky decision-making. Overconfidence may result in overestimation of one’s abilities or underestimation of opponents, task difficulty, or potential risks and can create illusions of control over events and immunity to risk (Block and Colvin, 1994).

In sports decision-making, overconfidence is often seen as egotism and a pervasive cognitive bias (Fogarty and Else, 2005). In soccer, referees are required to make quick and accurate decisions based on their understanding of the game rules, their observation of the situation on the field, and their own experience and intuition. However, overconfidence in their decision-making can lead to situations getting out of control. Referees must balance fairness and accuracy in making decisions regarding penalties while ensuring the smooth flow of the game. Overconfidence in their decision-making can exacerbate conflicts and lead to serious consequences (Erceg and Galić, 2014).

The systematic cognitive biases in DM described above, whether related to DMS or overconfidence, represent only a fraction of human cognitive fallacies. Behavioral experiments have shown that human thought processes exhibit systematic limitations and that judgment and DM are often only marginally rational (Hastie and Dawes, 2010). Various cognitive deficits, heuristic DM biases, and habitual thinking patterns influence most sports judgment and DM (Bennis and Pachur, 2006; Hepler and Feltz, 2012; Raab, 2012; Raab, 2017; Raab et al., 2019a,b; Ramanayaka et al., 2023). Similar systematic DM biases probably exist in soccer referee penalty DM, warranting an experimental exploration of the behavioral cognitive mechanisms underlying referee penalty DM. Hence, this study aimed to conduct a systematic experimental exploration of referee penalty DMS and overconfidence, which will not only help to correctly understand the referee’s task and the behavioral cognitive mechanisms involved in penalty DM but also enable the referee to improve the probability of rational DM in penalty DM behavior.

By comparing expert and novice referees, the study contributes to the understanding of how experience level affects decision-making processes and overconfidence, which can inform training programs for referees at different stages of their careers. The findings related to cognitive biases, such as overconfidence, in the context of sports officiating add to the broader literature on cognitive biases in high-stakes, time-pressured decision-making environments. The study contributes to the knowledge of how individual experience factors and match environment factors influence referees’ decision-making, building on previous research by providing a more comprehensive view of these influences. The research explores the interplay between System 1 (intuitive) and System 2 (analytical) in the context of sports officiating, contributing to the understanding of how these systems operate in high-pressure, real-time decision-making scenarios. Overall, this study enriches the body of knowledge surrounding sports officiating by providing empirical evidence on decision-making styles, the role of overconfidence, and the impact of expertise level, while also offering practical implications for training and performance enhancement.

So, the Purpose of this study: (I) Using soccer yellow-card foul videos as experimental materials, we explored the performance differences of soccer referees under different DMS when making decisions on whether to issue a yellow card or not. (II) We seek to assess the prevalence of overconfidence among referees of varying expertise levels by comparing penalty ACC, discrimination index (D), and self-confidence index (C). Additionally, we aimed to explore the effects of different penalty video types (VTs), DMS, and refereeing levels on overconfidence. Overconfidence holds significant implications for referees’ successive judgments and DM processes. This study explored the effect of overconfidence on different penalty VTs, DMS, and refereeing levels, as it served as a crucial reference point for referees’ ongoing DM endeavors. Hypothesis of this study: (I) Call performance might vary across different DMS for referees of varying expertise levels, and it might be influenced by both yellow and non-yellow card call VTs. Novice referees might exhibit better call performance in analytical decision-making (ADM) tasks compared to intuitive decision-making (IDM) tasks. Conversely, expert referees’ self-confidence in IDM may surpass that in ADM. Novice referees could potentially outperform IDM referees in terms of ADM discrimination, and the impact of different DMS on novice referees’ discriminative ability may exceed that of IDM referees. Moreover, different DMS may have a greater impact on novice referees compared to IDM referees. (II) There is evidence of overconfidence in referees’ decisions regarding soccer foul calls, and there might be an interaction between refereeing level and DMS. Expert referees might be more susceptible to overconfidence in IDM.

## Materials and methods

### Participants

Sixty male participants volunteered for this experiment and were divided into the expert group (*n* = 30) and the novice group (*n* = 30) based on their level of refereeing experience. In China, soccer referee grades are categorized into 5 levels from low to high, i.e., Level 3, Level 2, Level 1, National Level, and International Level. Each level has corresponding theoretical and practical examination standards. Generally speaking, undergraduates of soccer majors can get the qualification of level 3 referee, and graduates of master’s degree of soccer majors can get the qualification of level 2 referee. Enforcement of professional soccer matches [e.g., the Chinese Football Association (CFA) Division Two League] requires referees to be qualified as Level 1 referees or above.

The expert group comprised referees at national level 1 and above, including 9 national level referees and 21 national level 1 referees, affiliated with the Liaoning Provincial Football Association, Shenyang Football Association, and Changchun Football Association. In this study, we defined “expert” as a referee with level one referee standard or above and a referee with at least 5 years of experience in enforcing professional league matches. Those who met this criterion were included in the expert subject group. “Novices” were defined as graduate students or undergraduates who had level 3 referee standards or had experience in amateur soccer refereeing. Expert group participants were recruited through the coordination of the Chinese Football Association, recommended by the Liaoning Provincial Football Association, Shenyang Football Association, and Changchun Football Association, and the research group recruited by phone to confirm the participation of some active referees and non-active referees in the experiment. Two of them were active referees in the Chinese Football Association Super League, eight were active referees in the Chinese Football Association China League, and 20 had experience officiating in professional leagues such as the Chinese Football Association China League or the Chinese Football Association Division Two League, all with enforcement experience of more than 6 years. The subjects in the novice group were recruited from graduate and undergraduate students majoring in soccer at Shenyang Sports University, with some refereeing experience and a referee rating of national level 3 or below.

All participants completed a self-report questionnaire before the experiment that included demographic variables, health status, history of illness, history of brain injury, vision and correction, dominant hand, and experience of whether they had participated in a similar experiment. All participants reported good health, no history of genetic disease, no brain injury, no neurological disease, normal or corrected vision, and no prior relevant experimental experience. All participants were right-handed and provided informed consent before the experiment. They received modest compensation upon completion of the experiment.

As shown in Table 1, the average experience of participants in the expert group of this study in enforcing professional matches was 9.17 years (*M* = 9.17; *SD* = 2.25), while the average experience of participants in the novice group in enforcing amateur matches was 1.6 years (*M* = 1.6; *SD* = 0.72), and the average experience of participants in the expert group of the refereeing experience in del Campo et al. (2018) study was 10.25 years (*M* = 10.25; *SD* = 2.03), but in their study did not specify whether they were refereeing professional or amateur matches. In contrast, in Spitz et al. (2018) study, the average refereeing experience of the sub-elite referees was 12 years. Considering that the participants in the expert group in this study were experienced in enforcing professional matches, to become a referee enforcing professional matches, one has to accumulate many years in enforcing amateur matches (e.g., U-series youth soccer matches), therefore, referring to previous studies, the definition of the expert criterion in this study is still appropriate.

**Table 1:** Basic information of experimental participants.

	Age (*M* ± *SD*)	Referee level (*n*)		Enforcement experience (*M* ± *SD*)
Expert	34.77 ± 6.67	National level (9)	National level 1 (21)	9.17 ± 2.25
Novice	22.43 ± 5.06	National level 3 (12)	No level (18)	1.6 ± 0.72

### Instruments

For experimental video editing and the DM task system preparation, a desktop computer with the following specifications was utilized: a 22-inch color display, operating on a 64-bit system, equipped with a 2.3GHz processor. The screen resolution was set at 1024 × 768 pixels with a refresh rate of 60 Hz, and the viewing distance was maintained at 75 cm.

E-prime 3.0psychological experiment programming software was used for intuitive psychological experiment programming. It offered ease of operation through drag-and-drop video functionality, allowing for high customizability of stimulus video presentation and behavioral data collection. It also ensured millisecond-level temporal accuracy and was used for DM tasks and data collection.

Additionally, Adobe Premiere Pro 2020, a professional video editing software, was adopted for editing experimental videos. It facilitated adjustments in video duration, sound management, mirroring, and other necessary modifications.

### Materials

The editing process involved the following steps: (1) Interception of video clips. The slow-motion replays of suspected yellow card fouls (with a controlled ratio of 1:2 for yellow card to non-yellow card incidents) were intercepted from footage of the 2018 Fédération International de Football Association (FIFA) World Cup Russia matches, and the video was intercepted to obtain a total of 150 video clips. (2) Video framing point and video duration editing. The frame points of the video screen were determined, covering the duration from the beginning to the end of the player’s foul in the video. The video duration was adjusted to fall within the range of 1,000–2000 ms. (3) Video muffling processing. To minimize interference from sound in the videos for participants, the edited videos were muffled. (4) Final video compilation: a total of 150 videos were obtained to meet the specified requirements, including 50 videos of yellow card penalties and 100 videos of non-yellow card penalties.

The screening process involved the following steps: (1) preliminary screening. Shenyang Sports University 2 soccer national referee group assessed suspected yellow card fouls in slow motion according to yellow card rules. The score was divided into 4 probability levels: 100–75%, 75–50%, 50–25%, and 25–0%. Slow-motion replay videos scoring within the 75–50% range for showing a yellow card were retained as yellow card penalty stimulation videos, while those scoring within the 50–25% range were retained as non-yellow card penalty stimulation videos. After preliminary screening of the 150 edited videos, only those agreed upon by at least two referees proceeded. Ultimately, 106 videos passed preliminary screening, including 38 yellow card penalty videos and 78 non-yellow card penalty videos. (2) DM task system preparation: Using E-prime 3.0 psychological experimental programming software, the 106 videos were programmed into the DM task system. The system actively collected data on participants’ reaction time and correctness rate of DM.

The second screening process was as follows: 106 DM tasks were performed by 30 soccer-specialized college students from Shenyang Sports University. Videos with response times falling within 2,000 ms and correctness rates ranging between 60 and 90% passed the final screening. Consequently, a total of 100 videos met these criteria and were retained for further analysis.

Screening results were as follows: The final number of videos obtained was 100, with 10 designated for the practice phase and 90 for the formal experimental phase. Among these, 30 were yellow card penalty videos and 60 were non-yellow card penalty videos. For ADM stimulus videos, participants were required to respond to penalties within 2,000 ms of the end of video playback. The duration of video playback ranged from 1,000–2,000 ms, allowing participants a total response time of 4,000 ms. The ADM videos consisted of 30 non-yellow card penalty videos and 15 yellow card penalty videos. Similarly, for IDM stimulus videos, participants had to respond within 500 ms of the end of video playback. Slow-played videos were processed with a 2-fold fast playback. The video playback duration ranged from 500 to 1,000 ms, providing a response window of 1,500 ms. The IDM video consisted of 30 non-yellow card penalty videos and 15 yellow card penalty videos.

### Design

The experiment used a 2 × 2 × 2 three-factor mixed experimental design. Among the three independent variables, the between-subjects variable was Referee Level (RL: expert, novice), while the within-subjects variable 1 was DMS (ADM, IDM), and the within-subjects variable 2 was VT (yellow card foul, non-yellow card foul).

The dependent variables included decision-making accuracy (ACC), discrimination index (D), confidence (C), and overconfidence index (OC). Decision-making accuracy (ACC) represented the percentage of correct responses out of the total responses by experimental participants, including both correct responses to go and no-go stimuli. It reflected the proficiency level in judgmental DM tasks. Discrimination index (D) measured the experimental participant’s perception of the video stimuli, indicating their ability to correctly recognize target or non-target stimuli. Confidence (C) reflected the participants’ confidence in their judgment during DM tasks, ranging from 1 (not confident at all) to 10 (very confident). For the OC, following Koriat et al. (1980), if a participant exhibited high confidence levels (rated 8, 9, or 10 out of 10) for a question but answered incorrectly, it indicated overconfidence. The participant’s overconfidence scores for all questions were summed and averaged by dividing the total by the number of questions. A higher value indicated a greater degree of overconfidence.

Discrimination index (D) was calculated as follows: (1) When a participant was presented with a signal (treating the yellow stimulus video as a signal), it was categorized as a hit if the participant identified it as a signal, and a miss if mistaken as noise (treating the non-yellow stimulus video as noise). (2) When a participant was presented with a noise, it was categorized as a false alarm if the subject identified it as a signal, and it was correctly rejected if identified as a noise, as shown in Table 2.

**Table 2:** Calculation of the discrimination index D.

	Reporting (yellow card)	Reporting (non-yellow card)
Yellow card	12	3
Non-yellow card	3	27

To calculate the D, we first determine the probabilities of hitting and false alarms. Given hitting probability (PH) = 12/15 = 0.8 and false alarm probability (PF) = 3/30 = 0.1. Referring to the Probability of Z (POZ) conversion table, PH = 0.8 corresponded to a *Z*-score of 0.84, and PF = 0.1 corresponded to a *Z*-score of −1.28. Therefore, D = *Z*-score (hitting) – *Z*-score (false alarm) = 0.84 – (−1.28) = 2.12.

### Procedure

Before the experiment started, participants completed an informed consent form. The staff provided an overview of the experimental procedures and precautions and recorded participants’ basic information including name, contact details, years of officiating experience, and refereeing grade.

**Figure 1:**
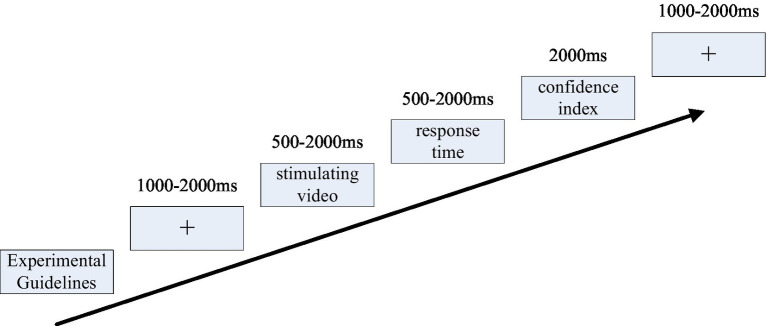
Experiment flowchart.

The experimental practice phase consisted of 10 trials, including 5 ADM videos (2 yellow cards and 3 non-yellow cards) and 5 IDM videos (2 yellow cards and 3 non-yellow cards). Following the practice phase, participants took a 5-min break to rest. During this break, they were informed of the experiment’s precautions and ensured comprehension before proceeding to the formal experiment. The experimental procedure is shown in Figure 1.

During the formal experiment phase, participants were briefed on the experimental procedure. The entire set of experimental videos was presented 90 times. These videos depicted slow-motion replays of fouls in soccer matches, each lasting between 500 and 2000 ms. Participants were tasked with determining whether each foul constituted a yellow-card offense and responding accordingly with designated keystrokes (“F” for yellow-card fouls and “J” for non-yellow-card fouls). In ADM, participants were given up to 2,000 ms to deliberate on each decision, followed by the determination of C within a 2,000 ms window after each decision. C used a 10-point Likert scale ranging from “not at all confident” to “very confident.” Conversely, in IDM, participants were required to make an immediate decision upon viewing the foul video, with the C determined after 2,000 ms. Responses in ADM were considered valid within 2,000 ms post-video playback, with any responses beyond this window marked as misses. Similarly, IDM responses were valid within 500 ms post-video playback. Trials appeared randomly with intervals ranging from 1,000 to 2,000 ms. The whole task duration was approximately 8 min, with 45 trials each for ADM and IDM, and alternation between expert and novice participants to mitigate sequential effects.

### Statistical analyses

SPSS Statistics 22.0 was used for data analysis, with DMS, VT, and RL serving as independent variables. Repeated measures analysis of variance (ANOVA) was conducted for ACC, D, C, and OC. The normality and homogeneity of variance for the aforementioned variables were assessed using the Shapiro–Wilk test and Levene’s test, respectively. Parametric tests were applied as the data met the assumptions of normality and homogeneity of variance (*p* > 0.05). A 2 × 2 × 2 repeated measures ANOVA was performed with group (expert, novice) as a between-subjects factor, and DMS (ADM, IDM) and VT (yellow card penalty, non-yellow card penalty) as within-subjects factors to investigate the effect of the independent variables on the dependent variable. Effect sizes in the repeated measures ANOVA were calculated as *η^2^* and a *p-*value of less than 0.05 was considered a significant difference.

## Results

A repeated measures ANOVA was conducted with DMS (ADM, IDM), VT (yellow card video, non-yellow card video), and RL (expert, novice) as factors, and the percentage of correct responses (ACC) as the dependent variable. The sphericity assumption was satisfied (*p* > 0.05). The results indicated a significant main effect of DMS, *F*(1,58) = 5.291, *p* = 0.025, *η*^2^ = 0.084, indicating that ADM correctness (*M* = 0.763, *SD* = 0.006) was significantly higher than IDM correctness (*M* = 0.747, *SD* = 0.006), supporting Hypothesis 1. The main effect of VT was not significant, *F*(1,58) = 0.291, *p* = 0.592, *η*^2^ = 0.005. However, a significant main effect of RL was observed, *F*(1,58) = 326.405, *p* < 0.001, *η*^2^ = 0.849, with expert referees demonstrating a higher accuracy in foul calls (*M* = 0.843, *SD* = 0.007) compared to novice referees (*M* = 0.666, *SD* = 0.007), as detailed in Table 3, which also supports Hypothesis 1.

**Table 3:** Decision-making style (DMS), VT, and RL on response correctness (ACC) for repeated measures ANOVA.

Source of variance	*df*	*F*	*p*	Partial *η^2^*
DMS	1	5.291	0.025	0.084
VT	1	0.291	0.592	0.005
RL	1	326.405	0.000	0.849
DMS × VT	1	0.425	0.517	0.007
DMS × RL	1	20.093	0.000	0.257
VT × RL	1	2.816	0.099	0.046
DMS × VT × RL	1	1.007	0.320	0.017

The interaction between DMS and RL was significant, *F*(1,58) = 20.093, *p* = 0.001, *η*^2^ = 0.000. Further simple effects analyses were conducted to explore this interaction. Among expert-level participants, there was no significant difference in the percentage of correct responses between ADM and IDM videos (ADM: *M* = 0.835, *SD* = 0.008; IDM: *M* = 0.851, *SD* = 0.009; *p* > 0.05). In contrast, novice-level participants showed a significant difference in the percentage of correct penalties depending on DMS (*p* < 0.05); specifically, the percentage of correct analytical penalties (*M* = 0.690, *SD* = 0.008) was significantly higher than that of intuitive penalties (*M* = 0.643, *SD* = 0.009), confirming Hypothesis 1 and indicating that different DMS only affect novice penalties. This finding is supported by the data presented in Table 3.

A 2 (DMS: ADM, IDM) × 2 (RL: expert, novice) repeated measures ANOVA was conducted with DMS as the within-subjects independent variable and RL as the between-subjects independent variable, using the C as the dependent variable. The test of sphericity was established with *p* > 0.05. The results showed a non-significant main effect of DMS [*F*(1,58) = 0.668, *p* = 0.417, *η^2^* = 0.011], which did not support Hypothesis 1. However, the main effect of RL was significant [*F*(1,58) = 79.018, *p* = 0.000, *η^2^* = 0.577], revealing that C of expert-level referees was significantly higher (8.629 ± 0.115) for foul calls compared to novice-level referees (7.180 ± 0.115), thereby supporting Hypothesis 1. The interaction between DMS and RL was significant [*F*(1,58) = 31.874, *p* = 0.000, *η^2^* = 0.577]. Subsequent simple effects analyses of this interaction, with RL tested separately at each of the two levels of DMS, revealed significant differences in self-confidence indices between expert-level participants when confronted with ADM versus IDM videos, *p* < 0.05. Specifically, experts showed a lower C for ADM (8.343 ± 0.141) compared to IDM (8.914 ± 0.120). Similarly, significant differences in self-confidence indices were found among novice-level participants when faced with different types of DM judgments (*p* < 0.05). Notably, self-confidence indices for ADM judgments (7.393 ± 0.141) were significantly higher than for IDM judgments (6.967 ± 0.120), confirming experimental Hypothesis 1. However, these findings suggest that different DMS have different effects on expert and novice participants, as shown in Table 4.

**Table 4:** Repeated-measures ANOVA of self-confidence index (C) in DMS penalties for different RL.

Source of variance	*df*	*F*	*p*	Partial *η^2^*
DMS	1	0.668	0.417	0.011
RL	1	79.018	0.000	0.577
DMS × RL	1	31.874	0.000	0.355

A 2 (DMS: ADM, IDM) × 2 (RL: expert, novice) repeated measures ANOVA was conducted with DM penalty type as the within-subjects independent variable and RL as the between-subjects independent variable, using the D as the dependent variable. A test of sphericity was established with *p* > 0.05. The results revealed a significant main effect of DMS [*F*(1,58) = 26.079, *p* = 0.000, *η*^2^ = 0.310]. Specifically, a significantly higher D for participants penalized for ADM (2.103 ± 0.056) than those penalized for IDM (1.829 ± 0.048), thereby supporting hypothesis 1. Additionally, a significant main effect of RL was observed [*F*(1,58) = 78.296, *p* = 0.000, *η*^2^ = 0.574], with a significantly higher D for foul calls by referee’s expert (2.362 ± 0.063) compared to novices (1.570 ± 0.063). Furthermore, the interaction between DMS and RL was significant [*F*(1,58) = 15.487, *p* = 0.000, *η*^2^ = 0.211]. Subsequent simple effects analyses of this interaction, with RL tested separately at each of the two levels of DMS, revealed that the difference in D (ADM: 2.394 ± 0.079; IDM: 2.331 ± 0.069) between expert-level participants when faced with ADM versus IDM was not significant (*p* > 0.05). However, novice-level participants showed significant differences in penalty D when faced with different DMS, *p* < 0.05. Specifically, D for ADM penalties (1.813 ± 0.079) was significantly higher than for IDM penalties (1.326 ± 0.069), confirming experimental hypothesis 1. These findings suggest that different DMS had an effect on the recognition of yellow card penalties only for novices, as shown in Table 5.

**Table 5:** Repeated-measures ANOVA of discrimination index (D) for different RL in different DMS.

Source of variance	*df*	*F*	*p*	Partial *η^2^*
DMS	1	26.079	0.000	0.310
RL	1	78.296	0.000	0.574
DMS × RL	1	15.487	0.000	0.211

A repeated measures ANOVA with a 2 (DMS: ADM, IDM) × 2 (VT: yellow card video, non-yellow card video) × 2 (RL: expert, novice) design was conducted, with DMS and VT as within-subjects independent variables and RL as the between-subjects independent variable, using OC as the dependent variable. The sphericity assumption was confirmed (*p* > 0.05). The results showed a significant main effect of DMS [*F*(1,58) = 6.253, *p* = 0.015, *η*^2^ = 0.097]. *Post hoc* comparisons indicated that the OC was significantly lower for ADM (23.883 ± 0.568) than for IDM (25.650 ± 0.410), supporting Hypothesis 1. Additionally, a significant main effect of VT was observed [*F*(1,58) = 11.551, *p* = 0.001, *η*^2^ = 0.166]. There was also a significant main effect of RL [*F*(1,58) = 95.421, *p* = 0.000, *η*^2^ = 0.622]. Expert-level referees exhibited a significantly lower OC in foul calls (23.883 ± 0.568) compared to novice-level referees (25.650 ± 0.410), as shown in Table 6, thus supporting Hypothesis 2.

**Table 6:** Repeated-measures ANOVA of DMS, VT, and RL on overconfidence index (OC).

Source of variance	*df*	*F*	*p*	Partial *η*^2^
DMS	1	6.253	0.015	0.097
VT	1	11.551	0.001	0.166
RL	1	95.421	0.000	0.622
DMS × VT	1	10.115	0.002	0.148
DMS × RL	1	6.136	0.016	0.096
VT × RL	1	65.038	0.000	0.529
DMS × VT × RL	1	2.216	0.142	0.037

The interaction between DMS and VT was found to be significant, *F*(1,58) = 10.115, *p* = 0.002, *η*^2^ = 0.148. Further analyses were conducted to explore the interaction between DMS and VT, with DMS tested at two levels of VT. Subsequent tests for VT revealed a significant difference in OCs between videos depicting yellow card penalties and those depicting non-yellow card penalties, *p* < 0.05. Specifically, the overconfidence indices for non-yellow card penalty videos (*M* = 24.767, *SD* = 0.550) were significantly higher than those for yellow card penalty videos (*M* = 23.000, *SD* = 0.640). However, the difference in overconfidence indices for IDM in both yellow and non-yellow card penalties (yellow penalty video: 25.550 ± 0.461; non-yellow penalty video: 25.750 ± 0.448) was not significant (*p* > 0.05). This finding supports Experimental Hypothesis 2, suggesting that only ADM affected yellow and non-yellow card penalties.

**Figure 2:**
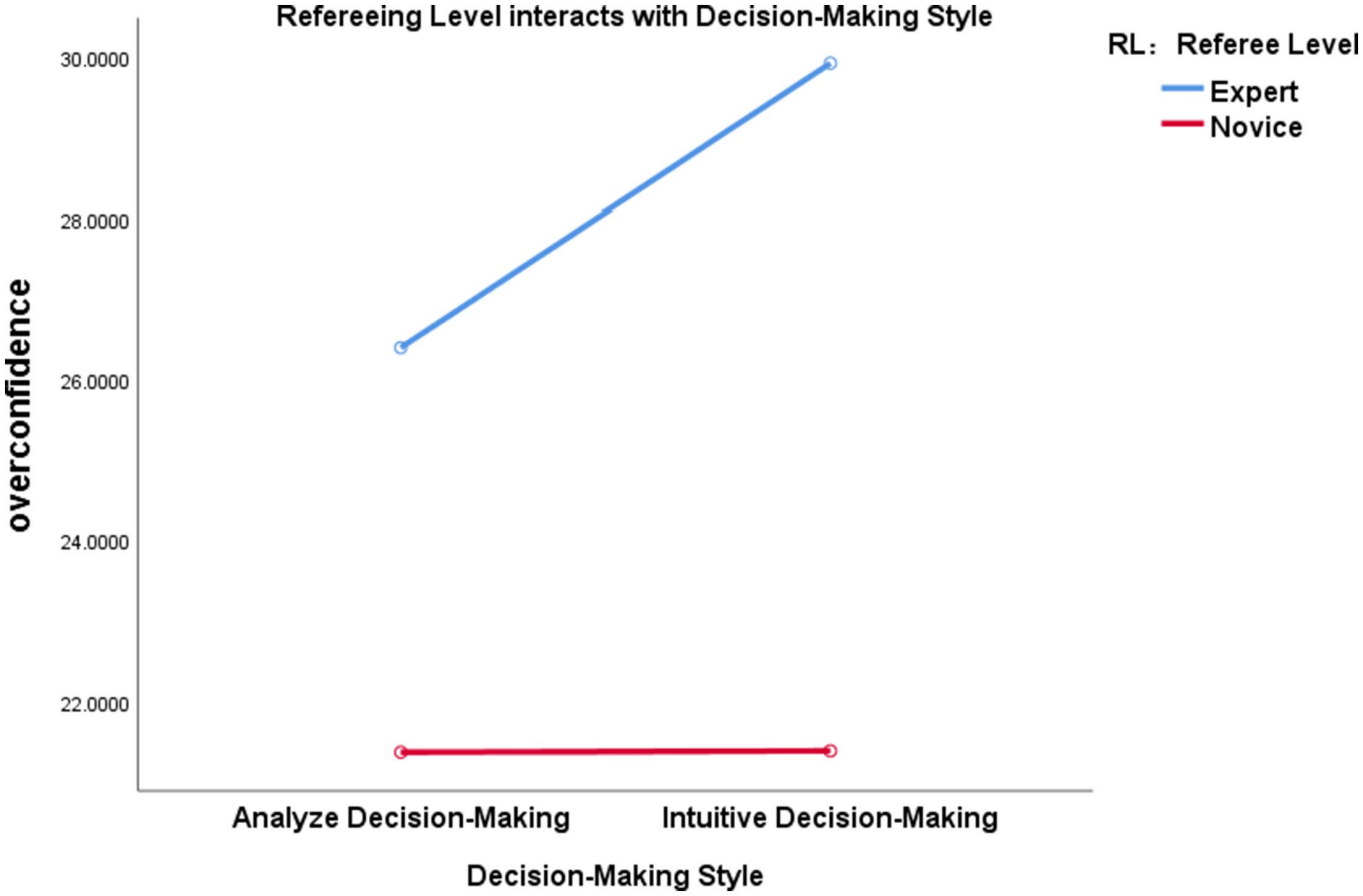
Referee level (RL) interacts with DMS.

The interaction between DMS and RL was found to be significant (Table 6), *F*(1,58) = 6.163, *p* = 0.016, *η*^2^ = 0.096. Further analyses were conducted on the interaction between DMS and RL, with RL tested at each level of DMS separately. It was found that the difference in overconfidence indices (ADM: 21.367 ± 0.804; IDM: 21.383 ± 0.579) between novice-level experimental participants exposed to ADM and IDM videos was not significant (*p* > 0.05). In contrast, a significant difference in OCs was observed among expert-level experimental participants when confronted with different DMS (*p* < 0.05). Specifically, OC for ADM (26.400 ± 0.804) was significantly lower than that for IDM (29.917 ± 0.579), indicating that different DMS only influence the overconfidence of expert sentencing, thus supporting Hypothesis 2 (Figure 2).

The interaction between VT and RL was found to be significant [*F*(1,58) = 65.038, *p* = 0.000, *η*^2^ = 0.529]. Further simple effects analyses were conducted on the interaction between VT and RL, with RL tested at each level of VT separately. Among expert-level experimental participants, there was a significant difference in overconfidence indices between yellow-card penalties and non-yellow-card penalties in videos (*p* = 0.002). Specifically, the OC for yellow-card calls (yellow-card call videos: 28.833 ± 0.568) was significantly higher than that for non-yellow-card calls (27.483 ± 0.493). Similarly, among novice-level experimental participants, there was a significant difference in OCs between yellow-card calls and non-yellow-card calls in videos (*p* = 0.000). OC for yellow-card calls (yellow-card call videos: 28.833 ± 0.568) was significantly greater than that for non-yellow-card calls (27.483 ± 0.493). Moreover, OC for yellow card penalty videos (19.717 ± 0.568) was significantly lower than that for non-yellow card penalties (23.033 ± 0.493). These findings suggest that OCs were affected by both yellow and non-yellow video DM penalties for both experts and novices.

## Discussion

Kahneman and Egan (2011), winner of the 2002 Nobel Prize in Economics, categorized the human DM thought process into IDM in System 1 and ADM in System 2. Both DM systems have controversial effects on DM performance. System 1’s IDM, characterized as “thermo-cognitive,” encompasses fast, parallelizable, automated, unconscious processes that require minimal cognitive resources, are associative, emotional, and effortless, and yield result-oriented, holistic, preconceived outcomes (Betsch and Kunz, 2008; Alós-Ferrer and Strack, 2014; Achtziger et al., 2015). In contrast, System 2’s ADM, termed “cold cognition,” involves controlled” or “thoughtful” processes that consume cognitive resources, are consciously monitored, and unfold slowly based on logical rules, cause-and-effect relationships, and a hierarchical, sequential, process-oriented approach (Shiloh et al., 2002; Shahar et al., 2015). While System 2’s ADM is presumed to exhibit fewer mistakes and greater accuracy than System 1’s IDM in general DM contexts, evidence from numerous studies suggests that humans can rely on intuition to swiftly and accurately navigate motor-related DM tasks across various contextual cues (Raab and Johnson, 2008; Schweizer et al., 2011; Hepler and Feltz, 2012; Collins et al., 2016; Raab et al., 2019a,b; Samuel et al., 2019). However, the fast contingency of IDM determines the probabilistic nature of DM performance, with speed prioritized over accuracy under conditions of time constraints and spatial compression. Drawing upon Klein et al.’s (2010) Recognition Primed-Decision (RPD) model DM in the domain of motion can be conceived as a three-stage recognition process: (I) simple match (recognizing a situation and associating it with the first adequate option), (II) diagnosing the situation (encountering an unfamiliar situation and requiring time to adapt a typical action), and (III) evaluating a course of action (assessing the relevance of the first option through mental visualization). Macquet’s (2020) literature review of RPD modeling in sports suggests that 60–81% of sports-related DM involves simple matching, 13–28% is associated with diagnostic situations, and 3–24% pertains to assessing a course of action. In addition, compared to athletes, rugby coaches experience less time pressure during DM and thus often engage in thoughtful or ADM (Collins et al., 2016). However, in soccer refereeing penalty DM, it remains to be empirically validated whether all penalty DM behaviors are IDM. Although fast and accurate decisions are conducive to the control and flow of the game, soccer refereeing DM does not invariably involve time-urgent, high-pressure DM; more often than not, accuracy processing is prioritized over speed processing. Furthermore, the demarcation between IDM and ADM is not always clear-cut, suggesting that DM in sports settings may occur along a continuum between IDM and ADM processes (Kahneman and Klein, 2009). Indeed, many sports DM processes may commence with intuition, which is subsequently validated and refined through analysis. From athlete DM (Ashford et al., 2021a,b; Hallé Petiot et al., 2021) to coach DM (Collins et al., 2016; Richards et al., 2016; Almeida et al., 2019) and referee DM (Kittel et al., 2021, 2023; Samuel et al., 2021), the Naturalistic Decision Making (NDM) approach suggests (Bossard et al., 2022) that athletes’ on-field decisions lean toward IDM, referees’ on-field decisions have equal importance of IDM and ADM, while coaches’ on-field decisions are dominated by ADM.

Despite previous research assuming that referees mostly rely on intuition to process information in contact situations (Plessner et al., 2009), the current study revealed that experimental participants were significantly more accurate in ADM compared to IDM in terms of penalty performance. This finding implies that ADM may play a crucial role in determining whether or not to issue a yellow card in DM situations. Regarding the value of C, no significant difference was observed between different DM systems. However, at different levels of expertise, participants’ performance in both DM systems remained consistent among expert-level participants, whereas novice-level participants showed a preference for ADM over IDM. It appears that experts can engage in both IDM and ADM concurrently, whereas novices rely more heavily on ADM in System 2. Analytical thinking can improve novice penalty performance under sufficient time. When faced with the binary task of classifying yellow card penalties, soccer referees must decide whether to issue a yellow card or administer a verbal warning in response to a foul situation, a task involving perceptual classification. Referees must consider visible cues to determine which criteria correspond to the card or no-card category. The accuracy of penalty DM depends on the referee’s ability to match cues with yellow card context encoding in long-term memory, with the current scenario processed through rapid retrieval and comparison with past yellow card episodes. Time constraints may prompt referees to respond via IDM rather than deliberate ADM processes. However, Schweizer et al. (2011) also argued that many DM tasks in soccer refereeing, such as discreet red and yellow card DM or offside DM, may necessitate more deliberate ADM over IDM.

Although Dijksterhuis et al. (2006) asserted in “Science” that IDM systems outperformed ADM systems, this assertion was not corroborated by the present study. Similarly, D, another performance metric, indicated that ADM outperformed IDM situations and was influenced by the level of refereeing. A simple effects analysis found that expert DM accuracy was not influenced by DMS, whereas novices were influenced by the decision-making system. This contradicted Hogarth and Schoemaker’s (2005) study, which suggested that subjects with rapid, intuitive characteristics in the DM process were generally more accurate compared to those with meticulous, analytical tendencies. In this study, ADM proved superior to IDM across all three dependent variables, and IDM did not demonstrate superior accuracy, speed, or performance characteristics in the soccer yellow card penalty DM task. It is obvious that in the formal yellow card penalty DM situation, there is no time pressure within the sub-500 milliseconds range, as seen in baseball batting (Chen et al., 2021). The process may take seconds or even tens of seconds from the foul occurrence to the card issuance, suggesting that the yellow card penalty DM paradigm may begin in IDM and be refined in ADM. In other words, both System 1 and System 2 could be involved in the DM process. Dual-system processing theory offers insights into information processing in yellow-card-foul DM situations, suggesting that the initial phase of penalty DM tends to favor contingent intuitive processing, wherein multiple features (e.g., cues) of the DM situation can be processed simultaneously in a very short period. Intuition is believed to rely on an extensive knowledge base in long-term memory acquired through associative learning (Betsch, 2008; Hogarth, 2008) and operates as a network of associations. IDM information processing resembles distributed parallel processing, while ADM processing more closely resembles serial processing. The serial computation of information processing in the yellow-card penalty DM context differs from distributed parallel computation, with advantages such as high accuracy, predictable results, and increased controllability in DM. It is clear that yellow-card penalty DM does not share the unique characteristics of typical sports DM. Yellow card penalty DM does not pursue speed priority as much as the most unique characteristics of time urgency and environmental coordination in ordinary sports DM. Instead, speed priority will be replaced by accuracy priority in yellow card penalty DM scenarios where the sports environment does not present urgent situations.

Many studies have shown that overconfidence in judgment and DM is widespread and frequent. It has been identified in everyday life activities. Overconfidence in DM has been frequently observed in the professional practices of numerous disciplines, such as doctors, lawyers, engineers, psychologists, and stock investors (Belsky and Gilovich, 2009). Surprisingly, research on overconfidence in the context of sports refereeing remains scarce. The overconfidence bias manifests when individuals’ confidence in their judgments exceeds the accuracy of those judgments. This traditional measure of overconfidence was used in the current study, revealing that both expert and novice referees, engaged in the yellow-card DM paradigm task, were influenced by their level of refereeing expertise, DMS, and the binary variable of whether a penalty was awarded or not.

In terms of referees’ yellow-card penalty DM self-confidence, expert referees were significantly more self-confident in awarding penalties than novice referees, consistent with previous research on team sport referees, where high-level referees had significantly greater self-confidence than low-level referees (Çar et al., 2022). In addition, refereeing level interacted with DMS, with simple effects analyses indicating that expert IDM self-efficacy was higher than ADM self-efficacy; conversely, novice referees showed the opposite pattern, suggesting that experts were more confident in their IDM accuracy, possibly indicating a preference for intuitive processing in System 1. In contrast, novices displayed greater confidence in ADM, suggesting a preference for this processing style. However, overconfidence did not yield completely consistent results with self-confidence. Contrary to common sense notions, experts in DM penalties were not significantly more overconfident than novices. Instead, novices were significantly more overconfident than experts, consistent with studies in sports betting. For instance, sportswriters and coaches, considered “experts,” did not predict the outcome of the second round of the 2002 FIFA World Cup more accurately than students, or “novices,” despite claiming to rely on information from extensive search and analysis (Andersson et al., 2003). Similar results were reported in another study on predicting teams for the 2006 FIFA World Cup tournament, where experts were not more successful than novices but showed considerable confidence in their predictions (Andersson et al., 2009). According to Griffin and Tversky’s (1992) explanation of the overconfidence bias, forecasters’ reliance on information could contribute to this bias. For example, teams playing at home have a higher probability of winning a game than when playing away (Nilsson and Andersson, 2010), suggesting that information about the match venue has predictive validity. Thus, knowledgeable and experienced decision-makers are more likely to be overconfident than those with less expertise because they have more knowledge and evidence upon which to base their judgments (Erceg and Galić, 2014). An additional explanation for overconfidence pertains to how individuals integrate evidence relevant to DM. According to Griffin and Tversky (1992), evaluating the consequences of a particular DM involves synthesizing various pieces of evidence. In most cases, two dimensions of evidence can be distinguished: the strength of evidence (extremity) and the weight of evidence (predictive validity) (Erceg and Galić, 2014). The interplay between these dimensions determines causality in DM. While predictive validity reflects the probability of an event occurring, overconfidence typically arises from the strength of the evidence (Erceg and Galić, 2014). The combination of these factors elucidates why experts tend to exhibit excessive caution in their DM. In situations with low predictability, each increment of expert knowledge enhances the strength of the evidence but does not influence its weight (i.e., predictive validity) (Erceg and Galić, 2014). Consequently, experts may possess superior judgment capabilities, yet the unpredictable nature of the situation hinders experience from accurately reflecting the accuracy of DM. As a result, experts’ overconfidence may be lower than that of novices, highlighting experts’ “fear of knowing.” The observed overconfidence among novices may reflect “ignorance without fear,” underscoring individual differences among referees (Avugos et al., 2021). The higher OC for expert IDM calls compared to ADM calls suggests that experts have greater confidence in their IDM, whereas novices are unaffected by DMS. Moreover, experts were more confident in awarding penalties than in withholding them, whereas novices displayed the opposite trend, indicating a bias towards self-protection among novices. Experts’ overconfidence in awarding penalties may also signify heightened assurance during critical moments. Conversely, novices’ overconfidence in not awarding penalties may serve as a form of self-protection.

### Limitations

The present study had some limitations. Firstly, IDM and ADM did not have a well-defined time cutoff point in the temporal processing process, and there existed a lack of consistent empirical evidence to delineate the transition from IDM to ADM in a sports context. In real-world referee DM situations where both systems may operate concurrently, disentangling the two in laboratory settings might pose a great challenge. Since this study draws upon prior sports DM studies, such as presentations of handball game contexts for 2000 ms (Tenenbaum et al., 1993), soccer game contexts for 2,000 ms (McMorris and Graydon, 1996), and basketball game contexts for 1,000 ms (Tenenbaum et al., 1999), and integrates insights from national-level referees, it is an unprecedented attempt to limit IDM to 1,500 ms and ADM to 4,000 ms. Additionally, slow-motion replay DM videos were played back at double the original speed, which was closer to the IDM requirements, i.e., rapid processing while disregarding intricate details. However, how exactly to separate IDM from ADM according to the specific DM task requires continuous research by researchers in the field of motion science. Furthermore, drawing from findings in other research domains (Calabretta et al., 2017), future studies in sports should explore the interplay between IDM and ADM across athletes, coaches, and referees (Bossard et al., 2022).

Secondly, there are two main paradigms in overconfidence measurement: confidence in binary decisions and interval prediction formats (Erceg and Galić, 2014). The classic method involves presenting participants with a series of questions, each offering two alternative answers. Participants are tasked with selecting the correct answer and providing their confidence level, with overconfidence inferred when the actual percentage of correct answers falls below the participant’s stated confidence level. The second paradigm requires participants to specify the range of intervals within which they believe the correct answer lies in a given DM scenario, along with their associated probability. For example, a participant might state, “I am 90% confident that the population of Zagreb, Croatia, falls between 700,000 and 1,000,000” (Erceg and Galić, 2014). Both paradigms can detect individuals who overestimate the accuracy of their judgments and thus quantify overconfidence. While the present study explored overconfidence in referees’ yellow card DM using the former paradigm, it is important to note that both paradigms rely on subjective reports from participants, which may be prone to inaccuracies and biases. Consequently, the measurement of overconfidence remains a subject of considerable debate. Therefore, future research should explore the use of more objective indicators to assess individual characteristics such as overconfidence.

Thirdly, several studies have demonstrated the significant impact of different video playback speeds on referees’ DM under controlled laboratory conditions (Put et al., 2016; Spitz et al., 2017, 2018; Jochim et al., 2018; Del Campo and Martín, 2020; Vater et al., 2024). Research on the impact of slow-motion replay and real-time video playback on referees’ DM regarding penalties has consistently revealed that referees tend to issue more severe penalties (including red or yellow cards) when viewing incidents in slow motion compared to real-time (Jochim et al., 2018; Spitz et al., 2018). Moreover, accuracy rates were found to be higher for decisions made using slow-motion replay (67%) compared to real-time viewing (56%) (Spitz et al., 2017). Contrarily, studies have shown that normal-speed playback resulted in higher ACCs for penalty decisions compared to 3× fast playback (Del Campo and Martín, 2020), and even slow-motion videos demonstrated higher DM accuracy than VR scenes (Vater et al., 2024). In contrast to the aforementioned findings, Put et al. (2016), in a study on offside penalty DM video simulation tasks, concluded that real-time and faster video conditions resulted in higher DM accuracy compared to slower video conditions. Video playback speed is an important variable, albeit with both positive and negative effects on penalty DM performance. In this study, high-definition (HD) playback video served as ADM material, while video playback at double the speed was used as IDM material. It is plausible that the confounding variable of video playback speed may have influenced the DM performance results, and future experiments may consider treating video playback speed as a covariate.

Lastly, because this study was a tightly controlled laboratory study, the entire experiment was tested in a laboratory setting. The referee’s decision-making was not affected by the numerous variables (influence factors) that occur in the real situation (i.e., during a match), such as the presence of the public, the position of the referees in the field, the home advantage, team ranking, etc. Therefore, while internal validity is guaranteed, external validity will inevitably be reduced, and the ecological validity of stimulus–response-type laboratory experimental studies has always been a pressing issue for sports scientists. The conclusions in this study are limited to laboratory situations, so extrapolating the findings of the study to refereeing decision-making in real soccer matches has to be approached with caution. In addition, the referee’s decision-making is now supported by video assistant referees (VARs), so some referee penalty decisions can be made later in the game after video viewing, and although communicating with VARs affects the flow and spectacle of the game, it does reduce the number of incorrect and missed calls in the game.

### Recommendations

The study on soccer referees’ yellow card decision-making has practical implications for training and performance improvement. Training programs should focus on analytical decision-making skills, encourage slower decision-making for accuracy, and include modules on managing overconfidence. Experience and Confidence: Expert referees have higher confidence in decisions. Novices should focus on building confidence through practice. Psychological Support: Referees need access to support and coping strategies for high-pressure situations. Feedback and Evaluation: Regular feedback helps referees understand their performance and areas for improvement. Education on Cognitive Biases: Training on cognitive biases can help referees be aware of decision-making pitfalls. Strategic Use of Intuition: Training should teach when to trust intuition and when to use a more analytical approach. Encourage Reflection and Learning: Debriefings and reflection sessions help referees learn from decisions and improve.

## Conclusion

This study confirmed that novice referees’ ADM penalty performance was superior to intuitive penalty performance in a soccer yellow card penalty DM task, while expert referees were not affected by DMS. In the yellow card offside penalty DM situation, both expert and novice referees showed overconfidence, and the degree of overconfidence was significantly higher in experts than in novices. Expert referees were more likely to be overconfident during IDM. They were more overconfident than non-yellow-card DM in awarding yellow-card DM, while novices were more overconfident than yellow-card DM in non-yellow-card DM situations. In conclusion, this study found that soccer referees were more likely to be overconfident in the yellow-card awarding DM task. The slower the referee’s DM, the more accurate it is, and the higher the referee’s level, the faster the referee’s DM, leading to increased confidence until overconfidence arises.

## Data availability statement

The raw data supporting the conclusions of this article will be made available by the authors, without undue reservation.

## Ethics statement

The studies involving humans were approved by the Shenyang University of Sport Ethics Committee. The studies were conducted in accordance with the local legislation and institutional requirements. The participants provided their written informed consent to participate in this study.

## Author contributions

HW: Writing – original draft, Writing – review & editing. CZ: Methodology, Writing – review & editing. ZJ: Methodology, Writing – review & editing. XL: Methodology, Writing – review & editing. LW: Writing – review & editing, Writing – original draft.
